# Engineered Human Induced Pluripotent Cells Enable Genetic Code Expansion in Brain Organoids

**DOI:** 10.1002/cbic.202100399

**Published:** 2021-09-13

**Authors:** Lea S. van Husen, Anna‐Maria Katsori, Birthe Meineke, Lars O. Tjernberg, Sophia Schedin‐Weiss, Simon J. Elsässer

**Affiliations:** ^1^ Science for Life Laboratory Department of Medical Biochemistry and Biophysics Karolinska Institutet 17165 Stockholm Sweden; ^2^ Center for Alzheimer Research Division of Neurogeriatrics Department of Neurobiology, Care Sciences, and Society Karolinska Institutet 17164 Stockholm Sweden

**Keywords:** amber suppression, brain organoids, genetic-code expansion, human induced pluripotent stem cells, non-canonical amino acids

## Abstract

Human induced pluripotent stem cell (hiPSC) technology has revolutionized studies on human biology. A wide range of cell types and tissue models can be derived from hiPSCs to study complex human diseases. Here, we use PiggyBac‐mediated transgenesis to engineer hiPSCs with an expanded genetic code. We demonstrate that genomic integration of expression cassettes for a pyrrolysyl‐tRNA synthetase (PylRS), pyrrolysyl‐tRNA (PylT) and the target protein of interest enables site‐specific incorporation of a non‐canonical amino acid (ncAA) in response to an amber stop codon. Neural stem cells, neurons and brain organoids derived from the engineered hiPSCs continue to express the amber suppression machinery and produce ncAA‐bearing reporter. The incorporated ncAA can serve as a minimal bioorthogonal handle for further modifications by labeling with fluorescent dyes. Site‐directed ncAA mutagenesis will open a wide range of applications to probe and manipulate proteins in brain organoids and other hiPSC‐derived cell types and complex tissue models.

## Graphical abstract

**Brain organoids** derived from human induced pluripotent stem cells (hiPSC) recapitulate key features of the developing brain and can model complex neurological disorders. Integrating the PylRS/PylT pair into the genome of hiPSCs allows efficient site‐directed incorporation of non‐canonical amino acids (ncAAs) and allows derivatization of neurons and brain organoids with an expanded genetic code. The method will make existing ncAA‐based strategies to probe and manipulate proteins in live cells accessible in hiPSC‐derived complex human disease models.

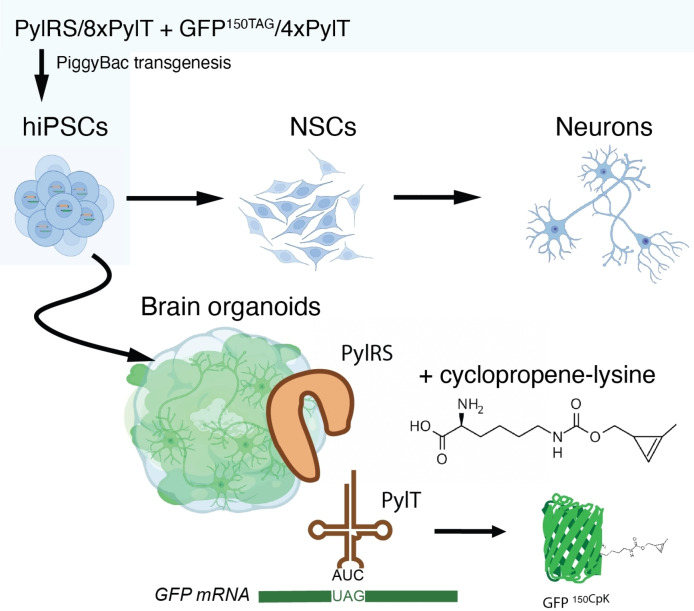

## Introduction

The systematic study of biochemical processes in neurodevelopment, neurodegeneration and other fields of human neuroscience is limited by availability of primary material and suitable model systems. Current knowledge is predominantly based on animal models or post‐mortem human brain. The informative value of the prevailing rodent models is limited by the evolutionary distance between humans and rodents.^[1]^ hiPSCs generated from patient samples and their differentiation to neurons and brain organoids could bridge the gap between animal models and clinical testing.[^2^, ^3^, ^4^] Since the first reprogramming of human fibroblasts to an induced pluripotent state with four defined transcription factors, OCT3/4, SOX2, KLF4, and c‐MYC,[^5^, ^6^] robust protocols for hiPSC generation from a wide variety of patient material have been established.^[7]^

Brain organoids recapitulate key features of the developing brain and can be derived from hiPSCs using defined protocols to model various brain regions and cell types like neurons, astrocytes, and oligodendrocytes.[^8^, ^9^, ^10^] Cerebral organoids have been successfully used to model neurological diseases, such as Alzheimer disease,^[11]^ Parkinson disease,^[12]^ microcephaly, autism spectrum disorders and Down syndrome.^[13]^

Non‐canonical amino acids (ncAAs) can introduce chemical functionalities not found in nature into a protein of interest. Genetic code expansion towards ncAAs requires a tRNA and an aminoacyl‐tRNA synthetase (aaRS), both orthogonal to the host cell (i. e. not interacting with the host tRNAs or aaRS enzymes). The pyrrolysyl aaRS/tRNA pair (PylS/PylT) from methanogenic archea is routinely used to suppress amber (UAG) stop codons and introduces a ncAA in response. This approach, termed amber suppression, has been used successfully in bacteria, yeast, mammalian cell culture, and animal models.[^14^, ^15^, ^16^, ^17^, ^18^]

ncAAs introduced via genetic code expansion cover a wide repertoire of chemical groups, including bioorthogonal handles, crosslinkable moieties and photocages.^[19]^ They have been shown to be invaluable tools for studying proteins important for neurobiological processes and pathophysiology, such as G‐protein coupled receptors[^20^, ^21^, ^22^, ^23^] and ion channels.[^24^, ^25^] ncAAs are particularly useful in applications where the protein under study cannot or should not be modified in a significant manner by larger protein modifications, such as fluorescent protein fusions or affinity tags. As an example, a ncAA has enabled fluorescent labeling of the Alzheimer disease‐associated amyloid β‐peptide (Aβ) sequence within the Aβ precursor protein.^[26]^

Genetic code expansion has been implemented in rat or mouse neurons through a variety of strategies.[^24^, ^27^] Using viral delivery, electroporation or lipofection, tRNA‐Synthetase/tRNA pairs were also introduced transiently into mouse brains or brain slices.[^28^, ^29^] However, no universal and efficient approaches exist to expand the genetic code of hiPSC‐derived human cultured neurons or complex organoids. Here we report the generation of hiPSCs with an expanded genetic code, enabling stable and efficient amber suppression in hiPSCs and neurons, as well as entire cerebral organoids derived from hiPSCs through *in vitro* differentiation. Genetic code expansion in cerebral organoids will facilitate implementation of the wide variety of ncAA‐based technologies developed for mammalian cells to relevant human model systems.

## Results and Discussion

**Figure 1:**
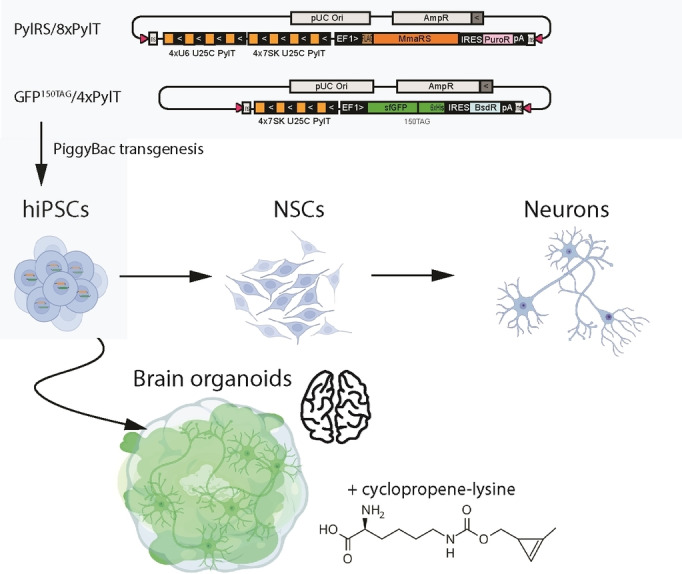
Integration of the amber suppression machinery in hiPSCs enables derivation of neurons and brain organoids with an expanded genetic code. Cells are co‐transfected with a PylRS expression vector and a PylT/sfGFP^150TAG^ reporter plasmid, carrying repeat cassettes for PylT expression, the PylS gene to produce PylRS and an amber stop‐codon containing GFP gene. hiPSCs are differentiated to NSCs, neurons and cerebral organoids. Culturing hiPSCs, NSCs, neurons or organoids in the presence of 0.2 mM cyclopropene‐lysine (CpK) leads to suppression of the amber stop codon and the production of GFP with a site‐specifically incorporated CpK moiety that can be subsequently derivatized using biorthogonal chemistry.

Stable genetic code expansion has been previously achieved in mouse embryonic stem cells using PiggyBac‐mediated transgenesis.^[16]^ We rationalized that establishing the PylRS/PylT system in hiPSCs would provide a route to ncAA incorporation via amber suppression in a wide variety of cell types that can be derived from hiPSCs. Below, we describe the generation of hiPSC line CTL07‐II‐AS with an expanded genetic code (Figure 1) from the hiPSC line CTL07‐II.^[30]^ CTL07‐II‐AS hiPSCs were differentiated into neural stem cells (NSC), neurons and cerebral organoids. We further demonstrate that amber suppression activity is maintained in mature neurons and neuronal organoid tissue.

### PiggyBac‐mediated integration of the orthogonal PylRS/tRNA pair

**Figure 2:**
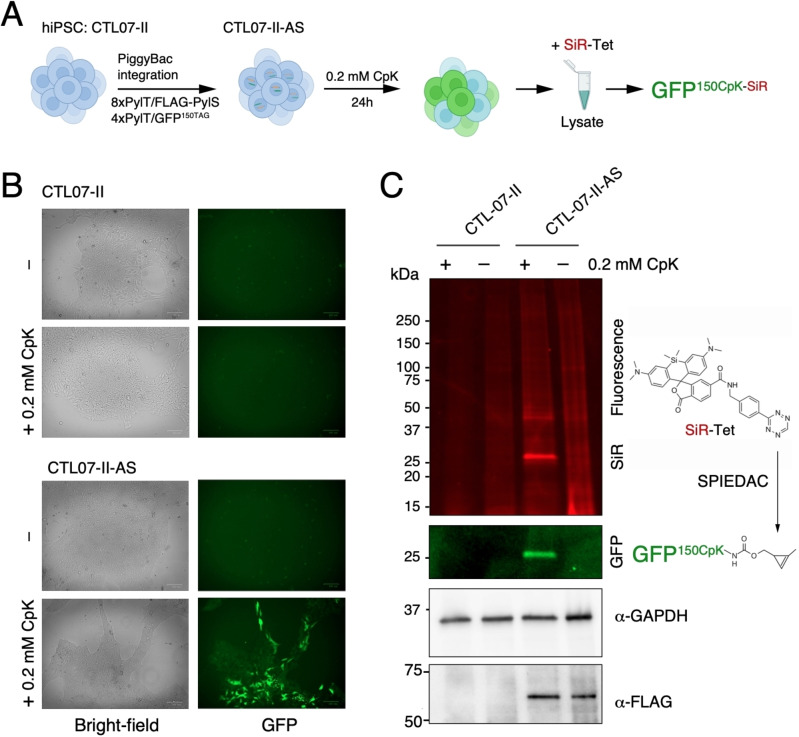
Stable amber suppression in hiPSCs. A) Scheme for generating CTL07‐II‐AS, a hiPSC line with expanded genetic code, CpK incorporation in the GFP^150TAG^ reporter and subsequent SPIEDAC fluorescent labeling with SiR‐Tetrazine. B) Brightfield and green fluorescence images of CTL07‐II and CTL07‐II‐AS. CTL07‐II‐AS cells that were incubated with 0.2 mM CpK for 24 h before imaging express GFP, while cells cultured without CpK and the parental cells do not show any fluorescence. C) SDS‐PAGE and fluorescent imaging of cell lysates of CTL07‐II and CTL07‐II‐AS, cultured in the presence or absence of 0.2 mM CpK for 24 h, incubated with 1 μM SiR‐Tetrazine to perform SPIEDAC labeling of CpK‐bearing proteins. Western blot shows FLAG‐PylRS expression in CTL07‐II‐AS cells. GAPDH was used as loading control.

To generate hiPSCs with an expanded genetic code, we used an updated two‐plasmid system for integrating amber suppression machinery and reporter[^16^, ^31^] using PiggyBac transposase (PBase). In the two targeting plasmids, expression cassettes include four or eight tandem repeats of PylT and are flanked by inverted repeats for transposition (Figure 1). The 8xPylT/PylS expression construct encodes FLAG‐tagged *Methanosarcina mazei* PylS, a puromycin resistance gene, and a total of eight PylT genes (four tandem repeats with U6 promoter and four tandem repeats with h7SK promoter). The 4xPylT/sfGFP^150TAG^ reporter plasmid encodes for GFP protein with an amber codon at position 150 (GFP^150TAG^), carries four tandem repeats of h7SK‐PylT and a blasticidin resistance gene (Figure 1). Since transfection efficiency via lipofection in hiPSCs is low, the 8xPylT/PylS plasmid was integrated in a first co‐transfection with PBase and stable transfectants were selected with puromycin. Then the 4xPylT/GFP^150TAG^ reporter plasmid was co‐transfected with PBase and double integrants were selected with puromycin and blasticidin, resulting in the polyclonal cell line CTL07‐II‐AS (Figure 2A).

### Efficient and selective non‐canonical amino acid incorporation in hiPSCs

To validate the generation of hiPSCs with an expanded genetic code, we incubated CTL07‐II‐AS cells with 0.2 mM cyclopropene lysine (CpK) for 24 h. GFP was expressed in the CTL07‐II‐AS cells only in the presence of CpK (Figure 2B), indicating that the GFP^150TAG^ stop codon is efficiently suppressed by the PylRS/PylT system. We also confirmed the expression of FLAG‐PylRS in CTL07‐II‐AS cells by western blot (Figure 2C). GFP levels were heterogeneous (Figure 2B), indicating that not all the cells in the population had strong amber suppression activity. Incorporation of CpK at GFP^150TAG^ was confirmed by performing strain‐promoted inverse electron‐demand Diels‐Alder cycloaddition (SPIEDAC) labeling of the cyclopropene moiety with silicon rhodamine (SiR)‐Tetrazine in cell lysate, subsequent SDS‐PAGE and fluorescent imaging (Figure 2C). A prominent band in the SiR channel overlapped with the fluorescent GFP band (Figure 2C, S1). Notably, the low abundance of background bands in the SDS‐PAGE after SiR‐tet labeling demonstrates that the amber codon is selectively suppressed in CTL07‐II‐AS despite the presence of amber stop codons in many endogenous genes (Figure 2C). ncAA incorporation into endogenous stop codons is thought to be disfavored by strong termination signals associated with terminal stop codons and quality control mechanisms that mitigate aberrantly elongated protein products.[^32^, ^33^, ^34^]

While this result hinted that the proteome of CTL07‐II‐AS hiPSCs was not collaterally affected by the amber suppression machinery, we also confirmed that expression of the pluripotency marker OCT4 was not affected by 24 h incubation with 0.2 mM CpK (Figure S2). Nevertheless, we note that applications of genetic code expansion in hiPSCs or derived cell lines need to consider inference from off‐target effects of amber suppression activity, and it will be important to optimize concentration and incubation time of ncAA to minimize such effects.

### Differentiation of hiPSCs with an expanded genetic code

**Figure 3:**
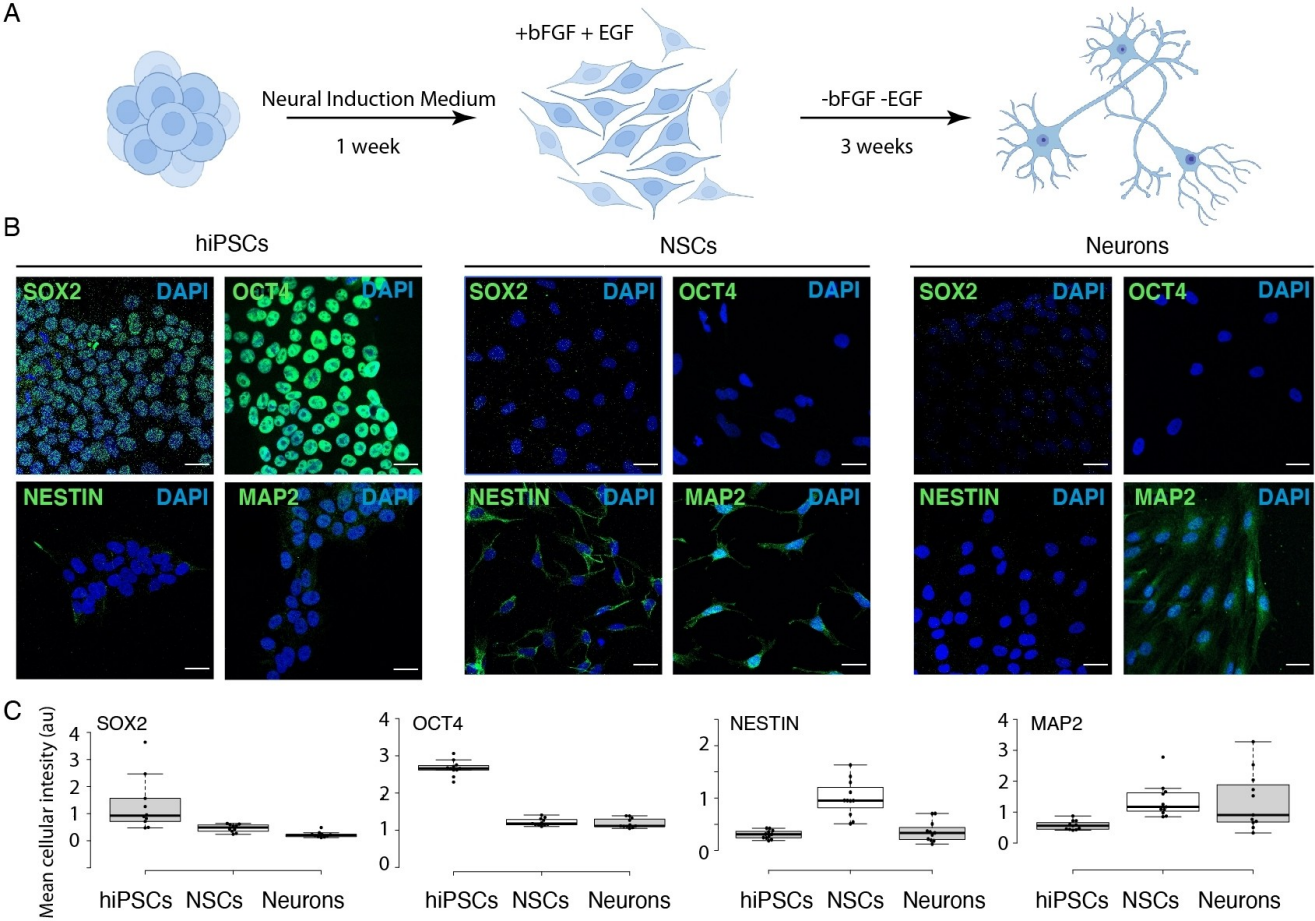
Neuronal differentiation of CTL07‐II‐AS. A) Scheme for differentiation of hiPSCs to NSCs and neurons. B) Immunofluorescence showing expression of stem cell markers SOX2 and OCT4 in hiPSCs, NSC marker NESTIN in NSCs and neuronal marker MAP2 in NSCs and 3‐week‐old neurons. Scale bars correspond to 30 μm C) Image quantification determining mean fluorescence intensity in each image (n=11) divided by the number of cells.

hiPSCs can be differentiated to neural stem cells (NSCs), a stable population of multipotent and self‐renewing progenitor cells that can be further differentiated to neurons, astrocytes or oligodendrocytes^[35]^ (Figure 3A). Differentiation to NSCs was performed within one week using serum‐free neural induction medium. Further differentiation to neurons was performed by removing bFGF and EGF stem cell growth factors for three additional weeks (Figure 3A). We followed the appropriate course of differentiation to mature neurons by immunofluorescent staining for the marker proteins OCT4, SOX2, NESTIN and MAP2 (Figure 3B). OCT4 is a marker for pluripotent stem cells whereas SOX2 is expressed throughout pluripotent and neural progenitors (Figure 3C). NESTIN is expressed in neural progenitors but not in mature neurons, while MAP2 is also expressed in mature neurons (Figure 3C). Hence, we could confirm the successful differentiation of hiPSCs with an expanded genetic code into neurons.

### Amber suppression in NSCs and neurons

**Figure 4:**
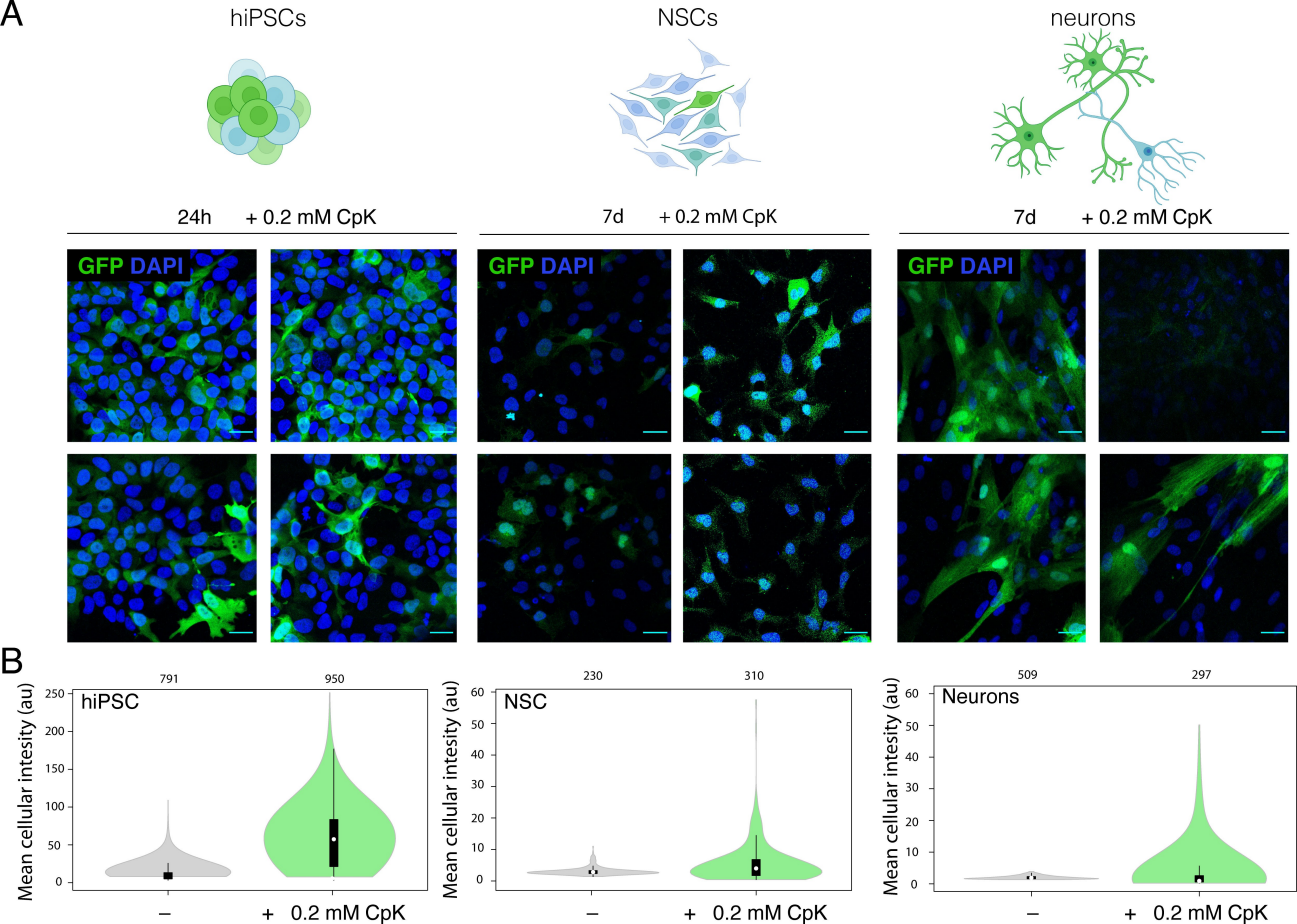
*In vitro* differentiation enables amber suppression in neurons. A) Four representative images from immunofluorescence microscopy of CTL07‐II‐AS hiPSCs, derived NSCs and neurons showing expression of GFP (green) and DAPI (blue). hiPSCs were cultured in the presence of 0.2 mM CpK for 24 h. NSCs and neurons were cultured in the presence of 0.2 mM CpK for 7 days. Scale bars correspond to 30 μm B) Image quantification showing the mean fluorescence intensity of each cell.

Genomic integration of the amber suppression machinery in pluripotent cells should in principle allow amber suppression in all derived cells and tissues. The EF1 promoter used here to drive mRNA expression and h7SK promoter for PylT are thought to be active in a cell‐type independent manner. While a 24 h incubation with 0.2 mM CpK was sufficient to elicit robust GFP^150TAG^ expression in hiPSC, even an extended incubation with CpK for 7 days elicited an intermediate GFP fluorescence in neurons and only low GFP signal in NSCs (Figure 4A, B). The expression of GFP was heterogeneous within the population in differentiated cells, as expected from the heterogeneous hiPSC starting population (Figure 4A, B). No GFP was produced in the absence of CpK in any cell type (Figure 4B, S3). These results demonstrate that stable incorporation of the PylS/PylT pair in hiPSCs enables genetic code expansion in derived terminally differentiated cells. Incorporation of CpK in neurons was further validated by labeling live neurons with SiR‐Tetrazine dye after incubation with 0.2 mM CpK for 7 days (Figure S4). Low GFP yield and limiting cell material did not allow us to further validate incorporation selectivity as done for hiPSC above. Overall, amber suppression efficiency appears to vary across different cell types, which could be a function of promoter strength for PylS and PylT genes, as well as the efficiency of translation termination competing with amber suppression in the respective cell type. Further, epigenetic silencing may lower transgene expression over time. Still, neurons had higher average GFP fluorescence than NSCs (Figure 4B), potentially owing to the fact that NSCs still divide and thus the GFP production is diluted continuously, whereas it can accumulate to higher levels in post‐mitotic neurons in the 7‐day incubation period with ncAA.

### Genetic code expansion in cerebral organoids

Having demonstrated amber suppression activity in terminally differentiated neurons from the stably integrated transgenes, we sought to derive more complex tissues from the CTL07‐II‐AS hiPSC line. Genetic code expansion in cerebral organoids would be particularly relevant for enabling site‐specific ncAA mutagenesis in model systems for neurological and neurodegenerative diseases, since polygenic diseases like autism spectrum disorders are known to affect multiple cell types and their connectivity and cooperativity in the brain.^[10]^

**Figure 5:**
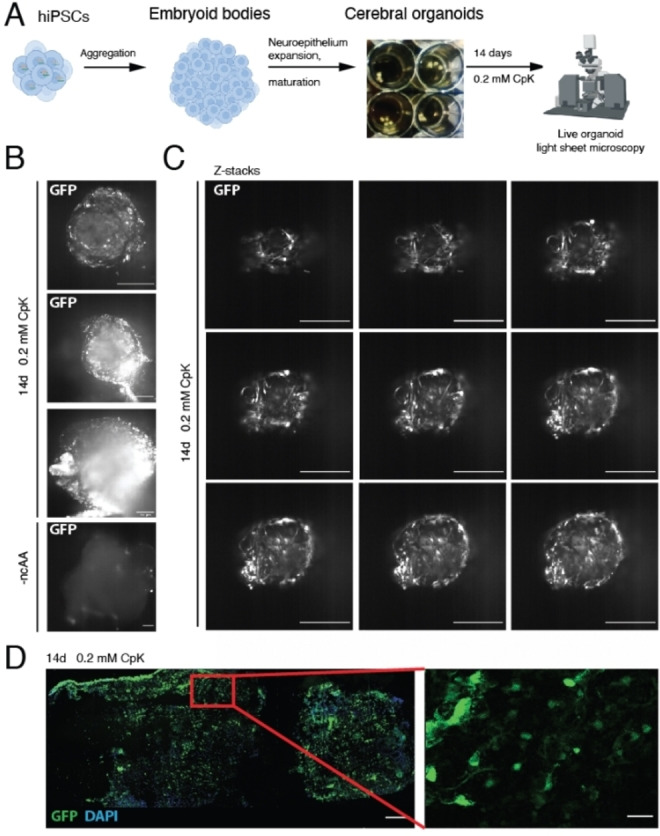
Stable amber suppression in cerebral organoids. A) Scheme for differentiation of hiPSCs into cerebral organoids. The organoids were differentiated for 40 days, cultured in the presence of 0.2 mM CpK for the last 14 days and imaged via light‐sheet microscopy. B) Live cell light‐sheet microscopy of cerebral organoids to detect GFP expression in organoids cultured in the absence or presence of 0.2 mM CpK. Selected planes are shown. Scale bars correspond to 250 μm. See Movie 1 for entire Z‐stack C) Light‐sheet microscopy Z‐stack images through the tip of the cerebral organoid cultured with 0.2 mM CpK. 250 μm scale bar. See Movie 2 and 3 for corresponding Z‐stack and 3D reconstruction. D) Immunofluorescent staining of fixed and cryosectioned organoid with a GFP antibody (green). DAPI is shown in blue. Scale bars correspond to 30 μm.

To this end, we differentiated CTL07‐II‐AS hiPSCs to cerebral organoids over the course of a 40‐day protocol, by first promoting the formation of embryoid bodies, which we differentiated further to neuroepithelial organoids and finally to mature organoids with cortical‐like regions (Figure 5A). The successful differentiation was confirmed by immunofluorescence microscopy of fixed organoid slices, in which we identified ventricular progenitor zones characterized by SOX2 expression and MAP2 neurons in the periphery (Figure S5). CTIP2, a deep‐layer subcortical projection neuron marker, was expressed in the outer layer of the organoids (Figure S5). We imaged live organoids, which were grown in the presence or absence of 0.2 mM CpK for 14 days, on a Zeiss Lightsheet Z.1 microscope (Figure 5A). We observed strong but heterogeneous GFP fluorescence in the presence of CpK across the organoid (Figure 5B, Movie 1). The particularly high GFP fluorescence in neuron‐like cells, and the lower fluorescence in neighboring cells made it possible to observe the neuronal morphology pervading the three‐dimensional organoid structure (Figure 5C, Movies 2, 3). We subsequently fixed the organoids for cryosectioning and immunofluorescence staining, and also observed the highest GFP intensity in neurites and soma of neurons (Figure 5D). The distinctive labeling of neurons was surprising considering that no neuron‐specific promoter was used, but consistent with our observation that cultured neurons accumulated more GFP than neural stem cells. In more internal regions, we also observed strong GFP fluorescence in tightly clustered cells forming luminal structures akin neuroepithelial rosettes (Figure S6, Movies 4, 5). These data demonstrate that genetic code expansion is possible in cerebral organoids with particularly high efficiency in differentiated neurons and luminal cells. The non‐canonical acid diffuses sufficiently well into the organoid to elicit efficient amber suppression also in deeper layers. Notably, genetic manipulation of mature organoids is difficult since viruses, lipofection or electroporation delivers DNA predominantly to the outermost layers. Thus, deriving organoids from hiPSCs with a stably expanded genetic code represents a key advantage in achieving amber suppression across the entire organoid. As discussed above, amber suppression efficiencies appeared to greatly vary with cell type, limiting the generality of our current approach and suggesting that amber suppression may be modulated by cell type‐specific properties, such as promoter strength and termination efficiency.

## Conclusion

In summary, we have created a hiPSC line with an expanded genetic code and demonstrated that genomically integrating the PylRS/PylT pair at the hiPSC stage allows derivatization of differentiated cells and complex tissues with an expanded genetic code. We anticipate that our approach of engineering hiPSCs will enable generation of many other cell types and tissues with expanded genetic code through established differentiation protocols. Translating the general approach demonstrated here by means of a GFP model protein to relevant functional proteins in specific cell types will necessarily require application‐specific optimization: we envision that the choice and optimization of promoters will be crucial to maximize ncAA incorporation efficiency in the desired cell type. For example, tissue‐specific promoters for the PylS gene could be exploited for restricting PylRS expression and thus amber suppression activity to a specific cell type or lineage. Our modular transgenesis approach also provides the opportunity to generate hiPSCs and derived cells or tissues stably expressing PylRS/PylT, combined with an alternative delivery of the protein of interest e. g. through lipofection or viral transduction.

We believe that human brain organoids with an expanded genetic code will provide a unique platform to study molecular mechanisms by using ncAAs to probe and manipulate proteins.

## Conflict of interest

The authors declare no conflict of interest.

## Supporting information

As a service to our authors and readers, this journal provides supporting information supplied by the authors. Such materials are peer reviewed and may be re‐organized for online delivery, but are not copy‐edited or typeset. Technical support issues arising from supporting information (other than missing files) should be addressed to the authors.

Supporting Information

Supporting Information

Supporting Information

Supporting Information

Supporting Information

Supporting Information
