# Supplementary material for: Engineered Human Induced Pluripotent Cells Enable Genetic Code Expansion in Brain Organoids
Source: Chembiochem. 2021 Sep 13;22(22):3208–13. doi: 10.1002/cbic.202100399 (PMC9290828; doi:10.1002/cbic.202100399)
Supplement: Supplementary file 1 — Supporting Information [file CBIC-22-3208-s002.pdf]

ChemBioChem

Supporting Information

Engineered Human Induced Pluripotent Cells Enable Genetic Code Expansion in Brain Organoids

Lea S. van Husen, Anna-Maria Katsori, Birthe Meineke, Lars O. Tjernberg, Sophia Schedin-Weiss,* and Simon J. Elsässer*

METHODS AND MATERIALS

SUPPLEMENTARY FIGURES 1-6

SUPPLEMENTARY MOVIE LEGENDS 1-5

UNCROPPED WESTERN BLOTS

PLASMID SEQUENCES

METHODS AND MATERIALS

DNA constructs

A sfGFP150TAG (superfolder GFP) reporter construct with four tandem h7SK-Mma PylT repeats has been described previously (Addgene #140015) [32]. The *M. mazei* PylT/RS expression plasmid B213 was generated by inserting four tandem h7SK-Mma PylT into the unique SpeI site in the published *M. mazei* 4xU6-PylT/RS expression plasmid [18]. All DNA constructs were verified by Sanger sequencing. Sequence of B213 is attached below and available at
https://benchling.com/organizations/elsasserlab_group

Non canonical amino acid and bioorthogonal labeling

The non canonical amino acid N-ε-[(2-methyl-2-cyclopropene-1-yl)-methoxy]-L-lysine (CpK, SiChem, SC-8017) was used for genetic code expansion. Stock solutions were prepared at 100 mM in 0.2 M NaOH/H2O, 15% DMSO. The GFP150CpK amber suppression reporter was labelled by SPIEDAC with Silicon rhodamine (SiR)-tetrazine (Spirochrome, SC-008). SiR-tetrazine 10 mM stock solution was prepared in DMF.

Cell Culture

Human induced pluripotent stem cell culture

hiPSCs (CTL07-II iPS) were purchased from the human iPS Core facility at Karolinska Institutet [30]. CTL07-II is a Male cell line, reprogrammed from fibroblasts, with normal 46 XY karyotype. Registration number: 2012/208-31/3.

Coating: 12-well plates were coated with 500 µl laminin-521 (Biolaminina, LN521) diluted 20x in PBS per well at 4℃ overnight or at 37℃ for 2 hours.

Medium: hiPSCs were cultured in Complete E8 medium (Gibco, A1517001) with Penicillin-Streptomycin (Sigma, P4333-100ml).

Culturing: hiPSCs were washed once with PBS (Sigma, A6964-500ML). 500 µl TrypleSelect (Gibco, 12563029) were added per well in a 12-well plate for 3 minutes at 37℃. Detached cells were transferred into a tube with 1 ml medium and centrifuged for 3 minutes at 300 g. Afterwards cells were resuspended in fresh medium and 10 µM ROCK Inhibitor (Millipore, Y-27632) was added. 75% of the medium was changed every day and cells were passaged every 3-4 days. Cells were imaged in a ZOE Fluorescent Cell Imager (BioRad).

Neural stem cell culture

Coating: 12-well plates were first coated with 0.02 mg/ml poly-ornithine (Sigma, #P3655-100MG) in PBS for 30 minutes at 37℃. After washing with PBS twice, culturing

plates were incubated with 2-4 µg/ml Laminin 2020 (Sigma, #L2020) in PBS at 4℃ overnight or at 37℃ for 4 hours.

Medium: To prepare 50 ml of Neural Expansion medium for culturing NSCs, 48.5 ml DMEM/F12+Glutamax (Gibco, #31331-028) were mixed with 500 µl N2 (Gibco, #17504-044), 500 µl Penicillin-Streptomycin, 50 µl B27 (Gibco, #17502-048), 10 ng/ml bFGF (Life Technologies, #CTP0261) and 10 ng/ml EGF (PreproTech, #AF-100-15).

Culturing: The cells were washed once with PBS before passaging. 500 µl Accutase (Sigma, A6964-500ML) were added to each well of the 12-well plates and incubated for 5-8 minutes at 37℃. Detached cells were transferred into a tube with 500 µl PBS and centrifuged for 4 minutes at 300g. The cells were resuspended in PBS and centrifuged one more time at 300g for 4 minutes. The supernatant was aspirated again, and the cells were resuspended in fresh Neural Expansion medium and added into the 12 well plates after removing the Laminin 2020 from the wells. ROCK inhibitor (Millipore, Y27632) was added to a final concentration of 5 µM. The medium was changed every second day and cells were split every 4-5 days.

Neurons

Coating: 12-well plates were first coated with 0.02 mg/ml poly-ornithine (Sigma, #P3655-100MG) in PBS for 30 minutes at 37℃. After washing with PBS twice, culturing plates were incubated with 2-4 µg/ml Laminin 2020 (Sigma, #L2020) in PBS at 4℃ overnight or at 37℃ for 4 hours.

Medium: To prepare 50 ml of medium for culturing neurons, 48.5 ml DMEM/F12+Glutamax (Gibco, #31331-028) were mixed with 500 µl N2 (Gibco, #17504-044), 500 µl Penicillin-Streptomycin and 500 µl B27 (Gibco, #17502-048).

Culturing: Neurons were cultured for 3-4 weeks. The medium was changed every second day and from day 14 on 2-4 µg/ml Laminin 2020 (Sigma, #L2020) was added to the medium.

Generation of stable cell lines

Stable cell lines were generated as published [31] with minor adaptations. In short, 100.000 hiPSCs (CTL-07-II iPS) were seeded in a 12-well plate 24 h before transfection. 5 µg DNA in 2:1 ratio (PiggyBac vector : pPBase vector) were diluted in 100 µl OPTI-MEM (Thermofisher, 31985070) before adding LT-1 (Mirus/Kem-EN-Tec-Nordic, MIR 2305). The transfection mix was incubated for 15 minutes to allow formation of transfection complexes, resuspended in 900 µl medium and added onto the cells. In the first step, the synthetase plasmid was integrated, and stable cell lines were generated through selection with 1 µg/ml Puromycin (VWR, CAYM13884-100). After selection the cells were passaged in presence of apoptosis inhibitor CloneR

(StemCell, #05888). In the second step the reporter plasmid (Addgene, # 140015) [32] was transfected, and the cells were selected with 200 µg/ml Blasticidin (Invivogen, ant-bl-10p) and 1 µg/ml Puromycin. After selection the cells were again passaged in presence of apoptosis inhibitor CloneR inhibitor (StemCell, #05888).

Differentiation of hiPSCs to NSCs and neurons

hiPSCs were differentiated to NSCs with Neural Induction Medium (Gibco, A1647801) according to the manufacturer protocol. In short, 24 h after splitting hiPSCs, at a confluency of around 20%, the medium was completely exchanged to 1 ml of the neural induction medium. Two days later, the medium is exchanged again. After 4 and 6 days, the medium was exchanged again, but 2 ml new medium were added into the 12-well plate. After 7 days of neural induction the cells were split into wells coated with poly-ornithine and L2020. NSCs were differentiated to neurons by removing bFGF and EGF from the medium and increasing B27 (Gibco, #17502-048) to 1:100.

Generation of cerebral organoids

Cerebral organoids were generated with help of the STEMdiff™ Cerebral Organoid Kit (StemCell, #08570) according to the manufacturer’s protocol. From day 25 to 40, 0.2 mM CpK was added to the medium.

Organoid light-sheet microscopy and immunofluorescence microscopy

Living organoids were imaged on day 40 with the Zeiss Light Sheet Z.1. Organoids were mounted in 1% low-melt agarose (LMA) in a glass capillary and the entire 3D volume of the organoid was imaged with a pixel width and height of 0.644 µm and a voxel depth of 2 µm. Afterwards organoids were fixed according to the previously published protocol from Lancaster and Knoblich [9]. In short, organoids were washed with PBS and fixed in 4% Formaldehyde (Thermofisher, 28906) at 4°C for 15 minutes. Afterwards organoids were washed 3 times with PBS and embedded in 30% sucrose (w/v) (Sigma, S0389-500G) solution at 4°C overnight. The next day, organoids were placed into a 7.5% gelatin/10% sucrose (w/v) embedding solution at 4℃, until the solution polymerizes. Organoids were frozen in a bath of Isopentane and stored at -80°C. Before Cryosectioning, the organoids were warmed to -20°C overnight. 20 µm slices of the organoids were cut via cryosectioning and washed with PBS-T at 37°C for 10 minutes to fully remove gelatin from slides. Afterwards slides were blocked in 5% Normal Donkey Serum in PBS-T for 1h and stained with different antibodies overnight at 4°C. Description of the antibodies and their dilution in 5% BSA in PBS-T can be found in the table below. The next day, slides were washed 3 times with PBS-T before the secondary antibody was added. After 2 hours incubation at room temperature, slides were washed 3 times in PBS-T for 30 minutes each. After cells were dried for 5 minutes Duolink® In Situ Mounting Medium with DAPI (Sigma, DUO82040) was

added. Slides were imaged using a Zeiss AiryScan800 laser scanning confocal microscope. Lasers and filters were chosen to illuminate at fluorophore excitation maximum. Images were processed using the software ImageJ. Illumination and gain settings were kept constant for all experiments.

**Antibody**	**Dilution**	**Supplier**
CTIP2	1:2000	Abcam (ab18456)
SOX2	1:2000	Rndsystems (MAB2018)
GFP	1:1000	Chromotek (PABG1-100)
MAP2	1:1000	Millipore (AB5622)
Donkey anti-Rabbit IgG, Secondary Antibody, Alexa Fluor 488	1:500	Life Technologies (A-21200)
Donkey anti-Mouse IgG, Secondary Antibody, Alexa Fluor Plus 647	1:500	Life Technologies (A-21236)

Immunofluorescence

Cells were washed with PBS and fixed in 4% Formaldehyde (Thermofisher, 28906) at 4℃ for 15 minutes. Afterwards cells were washed 3 times with PBS and permeabilized with 0.1% Triton X-100 for 15 minutes. Cells were blocked in 0.1 % BSA in 0.05% Triton-X-100 in PBS solution for 1 hour, before incubation with primary antibodies at 4℃ overnight. The source of the antibodies and their dilution in 0.1 % BSA in 0.05% Triton-X-100 in PBS can be found in the table below. The next day, cells were washed 3 times with PBS. Secondary antibodies were added for 2 hours, before the cells were washed again 3 times with PBS. After cells were dried for 5 minutes Duolink® In Situ Mounting Medium with DAPI (Sigma, DUO82040) was added. Slides were imaged using a Zeiss AiryScan800 laser scanning confocal microscope. Lasers and filters were chosen to illuminate at fluorophore excitation maximum. Images were processed using the software ImageJ. Illumination and gain settings were kept constant for all experiments. For the cell analysis, the software CellProfiler was used. To quantify the differentiation markers (SOX2, OCT4, NESTIN and MAP2) mean fluorescence intensity in each image, corrected for the number of cells, were measured. The mean intensity of GFP expression was measured for each cell.

Antibody	Dilution	Supplier
OCT4	1:500	Abcam (ab19857)
SOX2	1:2000	Rndsystems (MAB2018)
NESTIN	1:500	Millipore (MAB5326)
MAP2	1:1000	Millipore (AB5622)
GFP	1:1000	Chromotek (PABG1-100)
Alexa fluor 488 anti-rabbit	1:500	Life Technologies (A-21200)
Alexa fluor 555 anti-rabbit	1:500	Life Technologies (A-31572)
Alexa fluor 647 anti-mouse	1:500	Life Technologies (A-21236)

Bioorthogonal SPIEDAC labeling of neurons

Neurons were washed 3 times with medium and incubated for 2 h to remove excessive CpK. Afterwards the cells were cultured in the presence of 0.5 µM SiR-tetrazine (Spirochrome, SC008) for 15 min, before the cells were again 3 times washed and incubated for 1 h. After a last washing step, it was continued as described in the method section Immunofluorescence.

Western blots

Cells were lysed in N-Ripa Buffer (10 mM Tris-HCl (pH 7.6), 1 mM EDTA, 0.1% sodium dodecyl sulfate, 0.1% sodium deoxycholate, 1% Triton X-100, 5% Glycerol, 140 mM NaCl, 1x protease inhibitor) and sonicated for 20 cycles (30 sec on/30 sec off). Protein concentration was determined by BCA assay (Thermofisher, 23227). For lysate labeling 1 µM SiR-tetrazine (Spirochrome, SC008) was added to the lysate for 5 min. SDS PAGE sample buffer was added and samples were boiled briefly at 95℃. 25 µg protein were loaded in each well. Samples were loaded on 4–20% polyacrylamide Bis-Tris gels (BioRad, #4561096) and exposed for in-gel fluorescence at 460nm and 630 nm. The protein was transferred to nitrocellulose membranes and probed with FLAG-Antibody and GAPDH antibodies and imaged with the GE Healthcare ImageQuant LAS 500.

Antibody	Dilution	Supplier
FLAG	1:5000	Sigma (A8592)
GAPDH	1:10000	Millipore (AB2302)
GFP	1:500	ChromoTek (pABG1-100)
OCT4	1:500	Abcam (ab19857)
H3	1:15000	Active Motif (39763)
anti-mouse HRP	1:10000	Biorad (Cat#1721011)
anti-rabbit HRP	1:10000	Biorad (Cat#1721019)
anti-chicken IgY (H+L) HRP	1:10000	Invitrogen (A16054)

SUPPLEMENTARY FIGURES

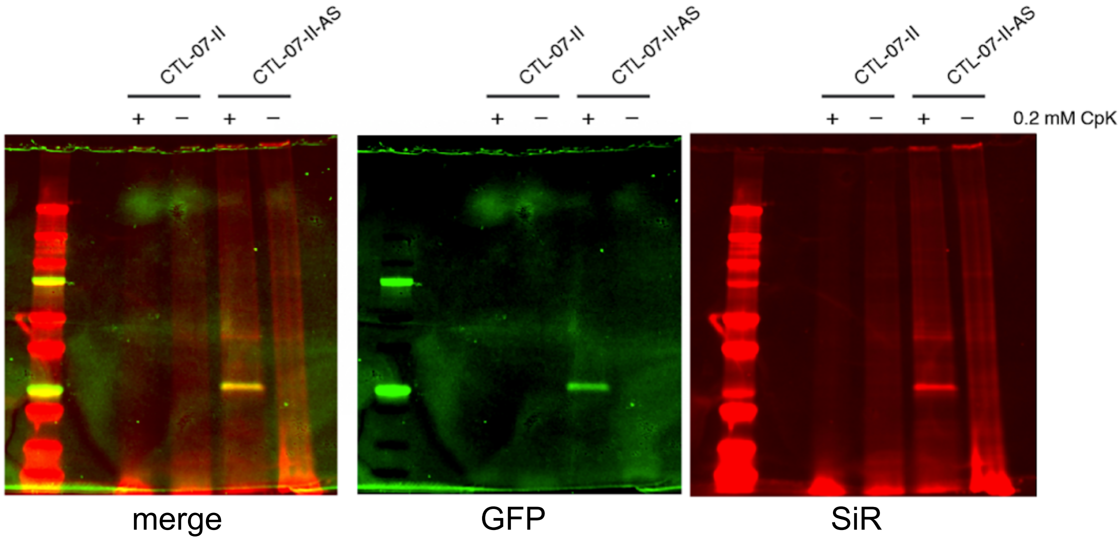

Supplementary Figure S1: Bioorthogonal SPIEDAC labeling of lysate from CTL07-II hiPSCs and CTL07-II-AS hiPSCs. Cells were cultured in the presence of 0.2 mM CpK for 24 h (+) or without CpK (-). Cell lysate was incubated with SiR-Tetrazine for 5 min before it was separated by SDS-PAGE and imaged for in-gel fluorescence using green (GFP) and red (SiR) channels.

Supplementary Figure S2

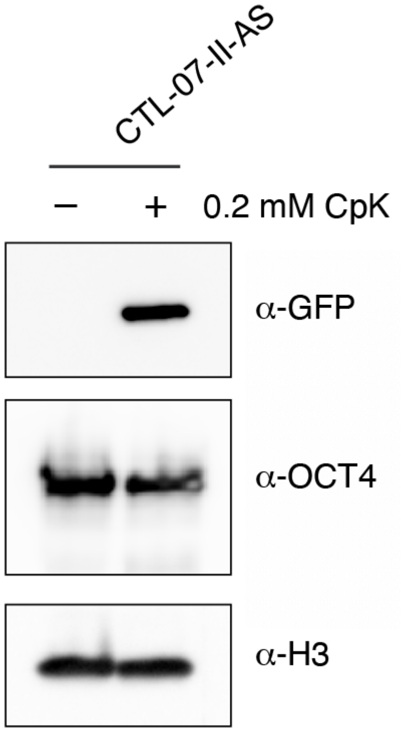

Supplementary Figure S2: Western blots from cell lysates of CTL07-II-AS showing expression of GFP when cultured in the presence of 0.2 mM CpK for 24 h. OCT4 was expressed in cells cultured in the presence and absence of CpK. H3 was used as loading control.

Supplementary Figure S3

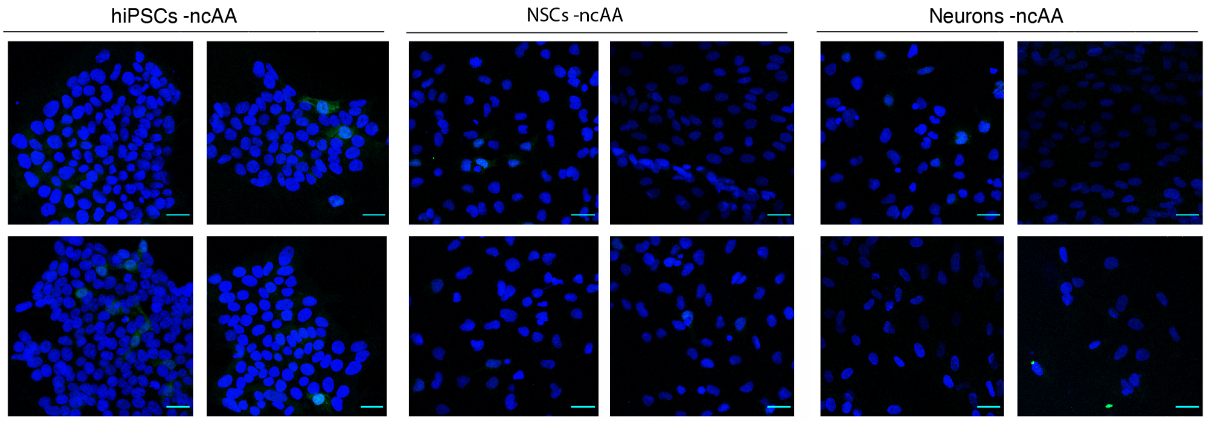

Supplementary Figure S3: Four representative images from immunofluorescence live cell microscopy of CTL07-II-AS hiPSCs, derived NSCs and neurons showing expression of GFP (green) and DAPI (blue), cultured in the absence of ncAA.

Supplementary Figure S4

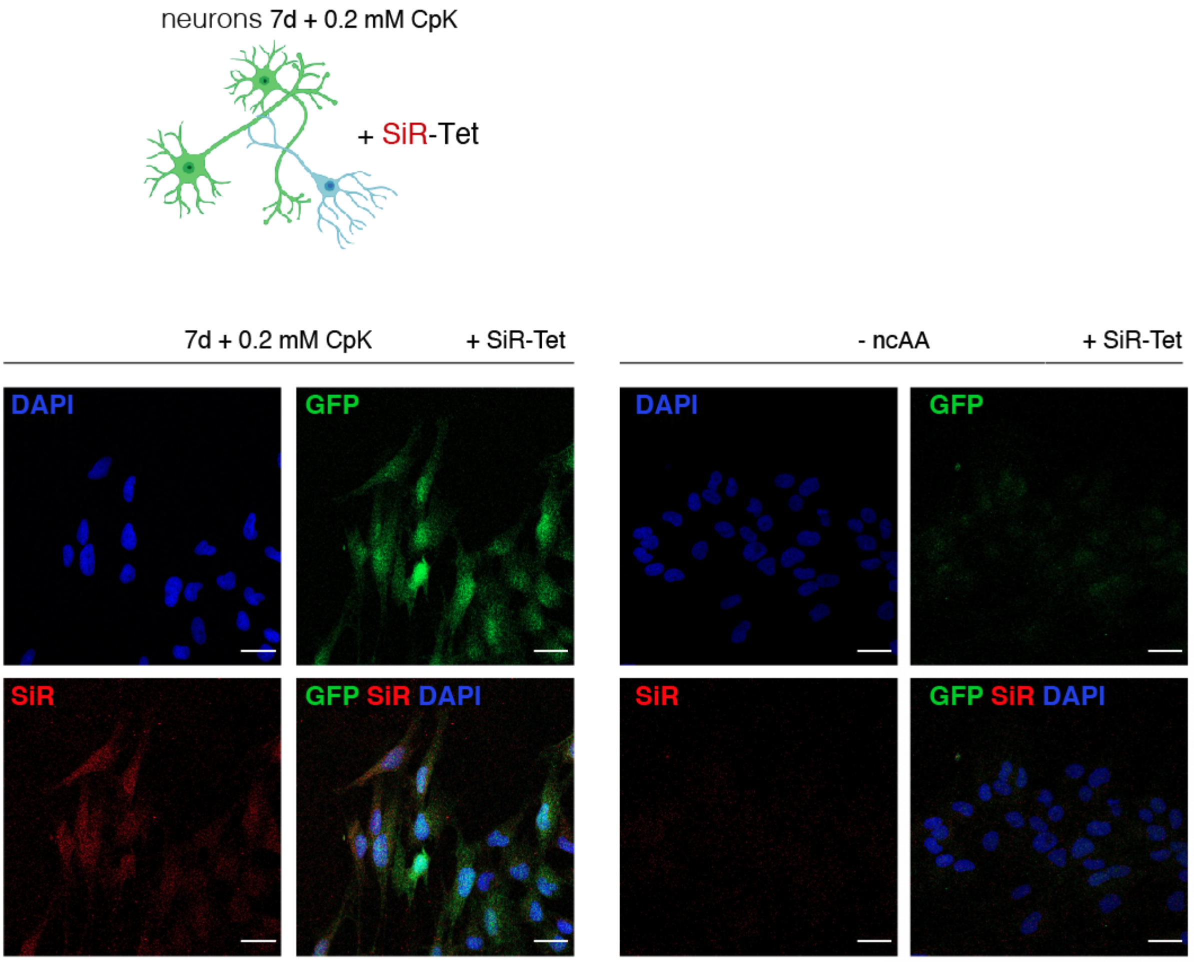

Supplementary Figure S4: Representative images of SPIEDAC labeled 3 week old neurons. Neurons were cultured in the presence or absence of 0.2 mM CpK for 7 days, before cells were incubated in 0.5 µM SiR-Tetrazine dye for 15 min before fixation. GFP expression is shown in green, SiR-Tetrazine labeling in red and DAPI in blue. Scale bars correspond to 30 µm.

Supplementary Figure S5

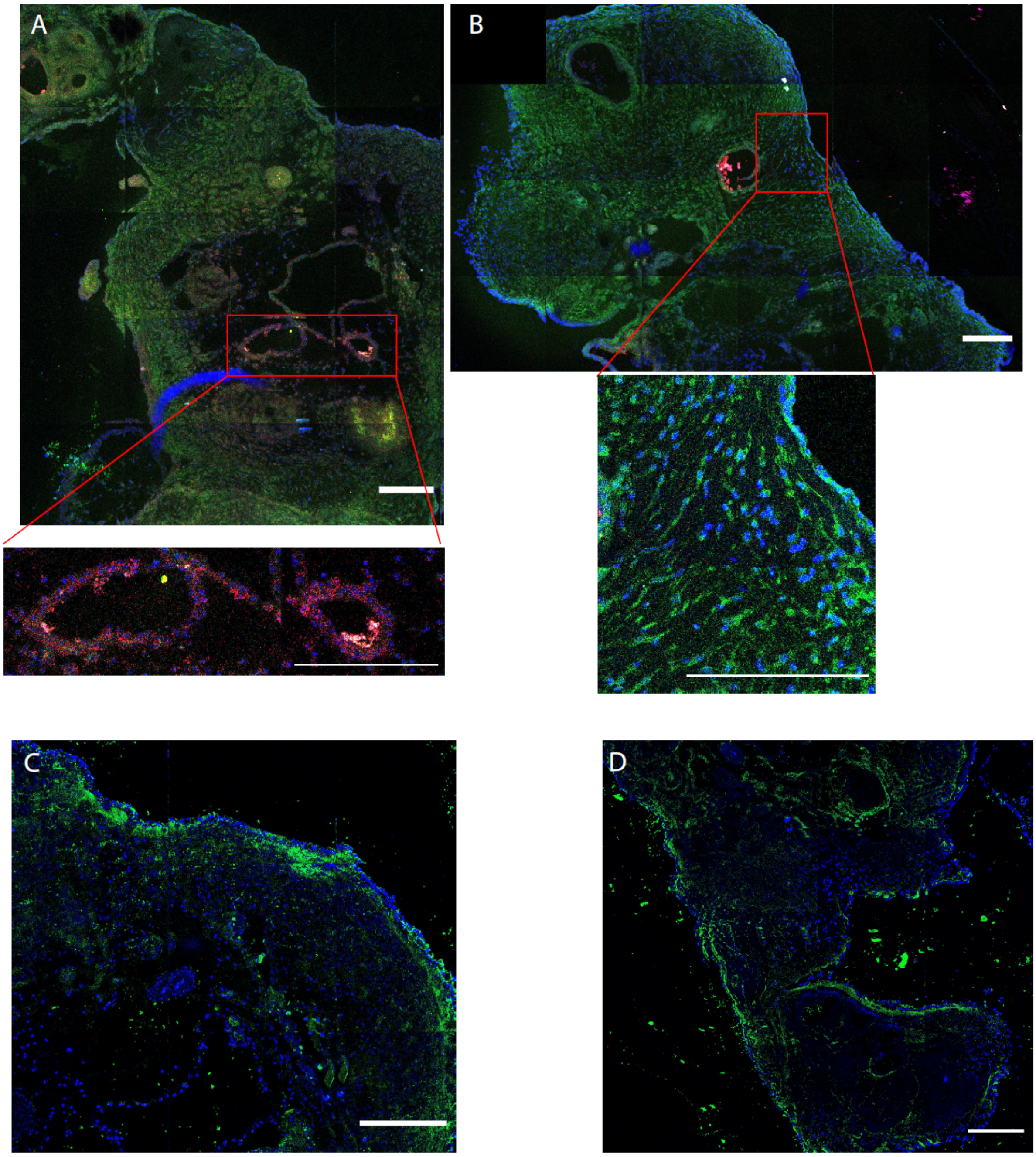

Supplementary Figure S5: Characterization of cerebral organoids generated from CTL-07-II-AS hiPSCs and differentiated for 40 days. A) DAPI (blue) and SOX2 (red) co-staining highlights rosette-like progenitor compartments around the ventricles. B) DAPI (blue) and MAP2 (green) show successful differentiation to neurons. C-D) DAPI (blue) and CTIP2 (green) staining shows deep-layer cortical neurons on the surface of the cerebral organoids.

Supplementary Figure S6

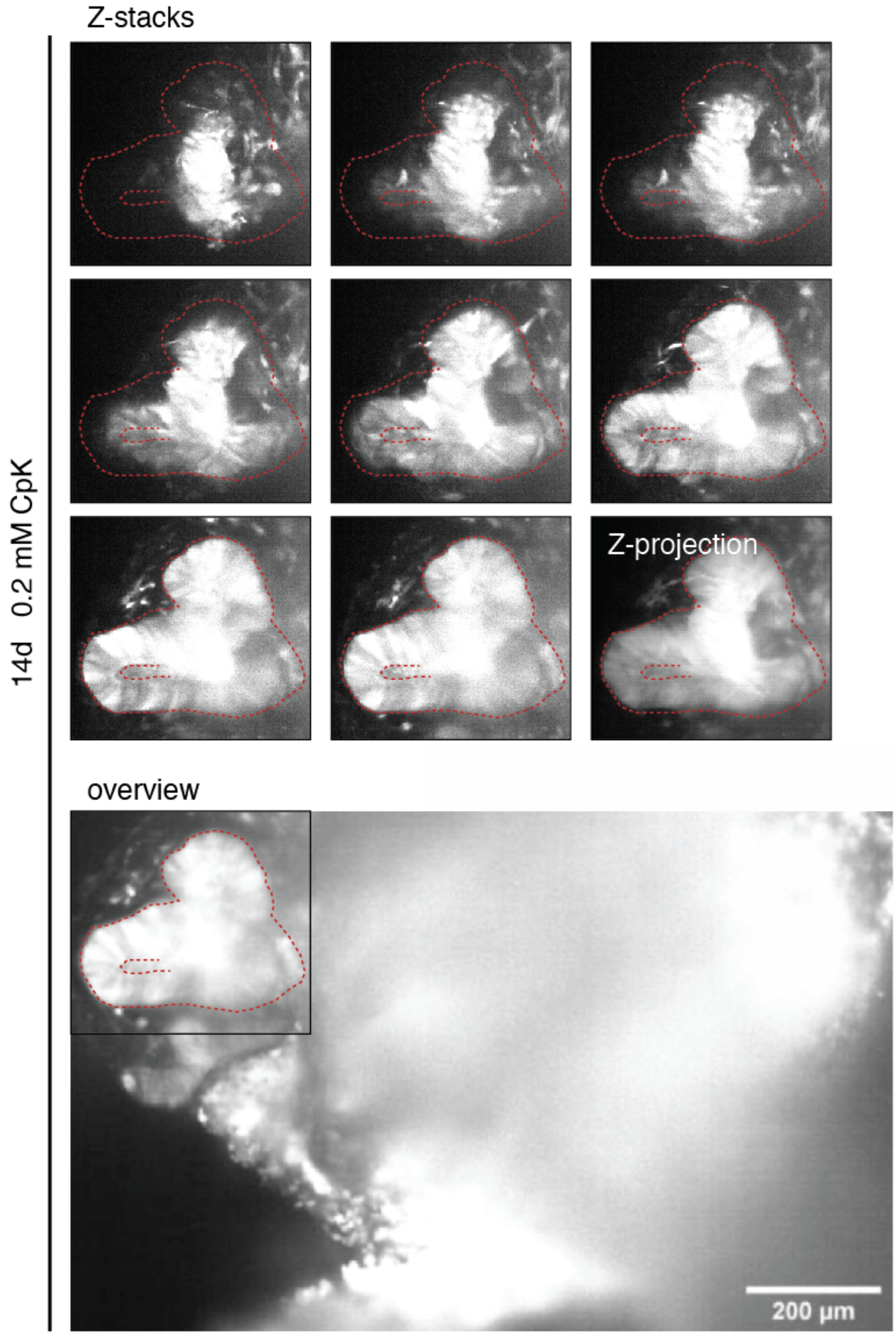

Supplementary Figure S6. Stable amber suppression in cerebral organoids. Live cell light-sheet microscopy of cerebral organoids to detect sfGFP expression in organoids cultured with 0.2 mM CpK Scale bars correspond to 200 μm. Luminal neuroepithelial structure with high GFP fluorescence is highlighted in red. See also entire Z-stack (Movie 4) and 3D reconstruction (Movie 5).

Supplementary Movie 1: Z-stack, GFP fluorescence, cerebral organoid

Live cell light-sheet microscopy (GFP channel) of a cerebral organoid cultured with 0.2 mM CpK.

Supplementary Movie 2: Z-stack, GFP fluorescence, tip of cerebral organoid

Live cell light-sheet microscopy (GFP channel) of the tip of a cerebral organoid cultured with 0.2 mM CpK.

Supplementary Movie 3: 3D projection, GFP fluorescence, tip of cerebral organoid

3D reconstruction from live cell light-sheet microscopy (GFP channel) of the tip of a cerebral organoid cultured with 0.2 mM CpK.

Supplementary Movie 4: Z-stack, GFP fluorescence, luminal cluster

Live cell light-sheet microscopy (GFP channel) of a luminal rosette within a cerebral organoid cultured with 0.2 mM CpK.

Supplementary Movie 5: 3D projection, GFP fluorescence, luminal cluster

3D reconstruction from live cell light-sheet microscopy (GFP channel) of a luminal rosette within a cerebral organoid cultured with 0.2 mM CpK.

UNPROCESSED WESTERN BLOT IMAGES

Figure 2

anti-FLAG M2 (SIGMA F3165), HRP

anti-GAPDH (Millipore AB2302), HRP

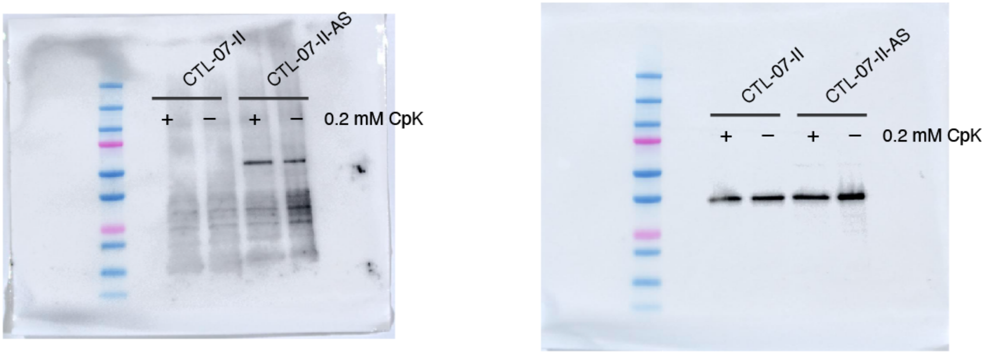

Supplementary Figure 2

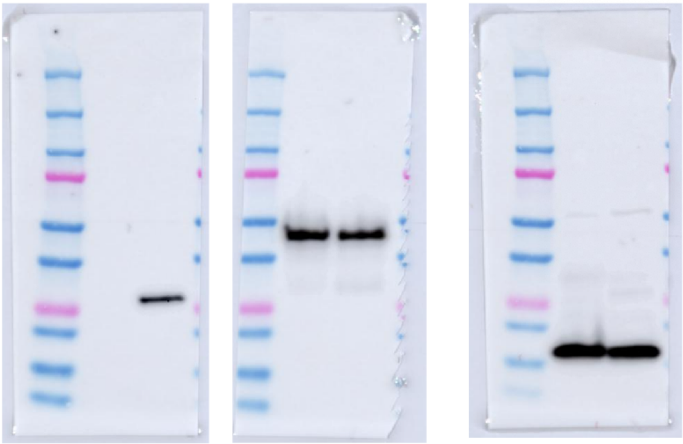

α-GFP α-OCT4 α-H3

Western blots from cell lysates of CTL07-II-AS showing expression of FLAG in comparison to the parental CTL07-II cells.

Western blots from cell lysates of CTL07-II-AS showing expression of GFP when cultured in the presence of 0.2 mM CpK for 24 h. OCT4 was expressed in cells cultured in the presence and absence of CpK. H3 was used as loading control.

Plasmid Sequence B213

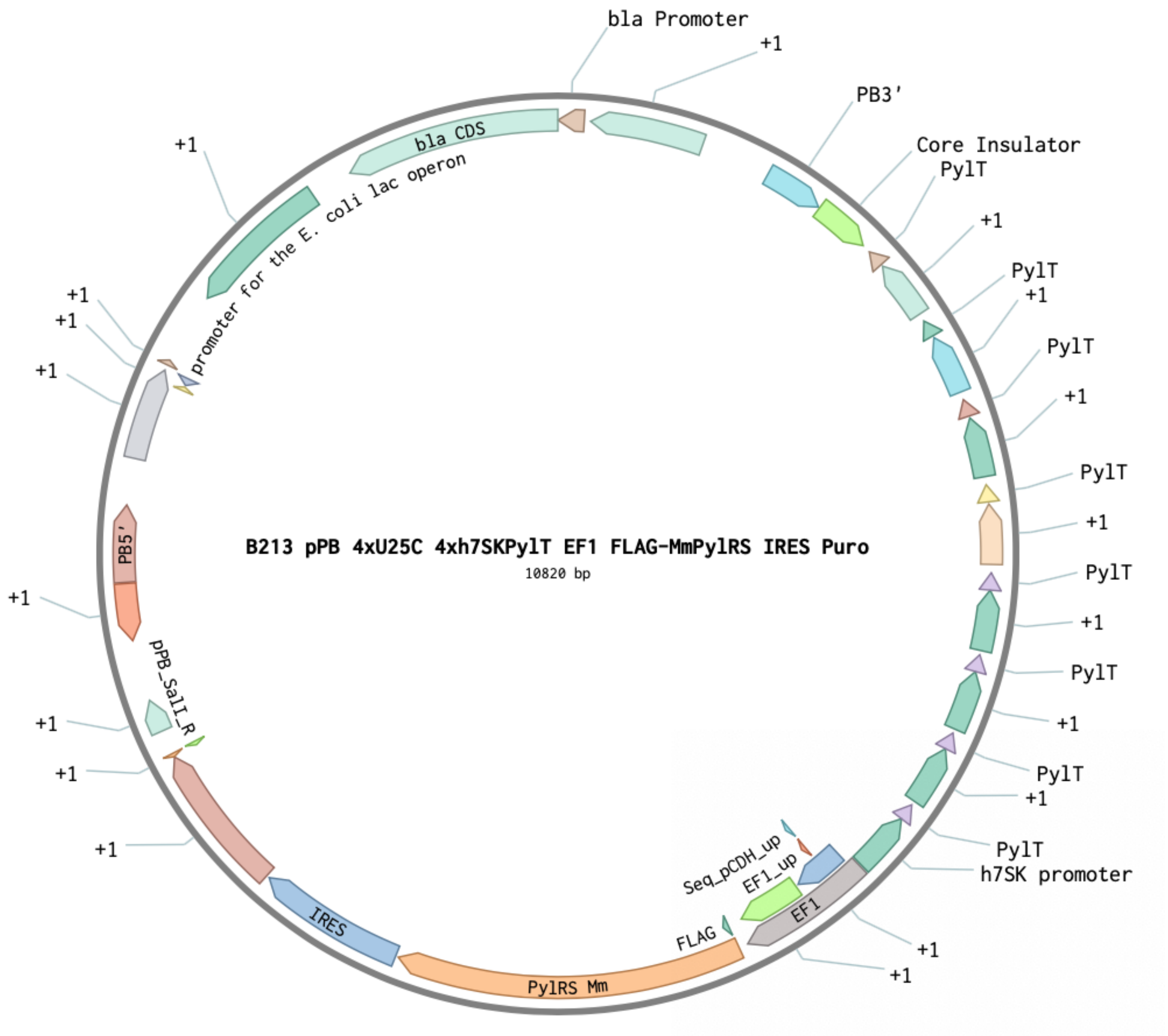

>B213 pPB 4xU25C 4xh7SKPylT EF1 FLAG-MmPylRS IRES Puro
ACTCTTCCTTTTTCAATATTATTGAAGCATTTATCAGGGTTATTGTCTCATGAGCGGATACATATTTGAATGTAT
TTAGAAAAATAAACAAATAGGGGTTCCGCGCACATTTCCCCGAAAAGTGCCACCTAAATTGTAAGCGTTAATATT
TTGTTAAAATTCGCGTTAAATTTTTGTTAAATCAGCTCATTTTTTAACCAATAGGCCGAAATCGGCAAAATCCCT
TATAAATCAAAAGAATAGACCGAGATAGGGTTGAGTGTTGTTCCAGTTTGGAACAAGAGTCCACTATTAAAGAAC
GTGGACTCCAACGTCAAAGGGCGAAAAACCGTCTATCAGGGCGATGGCCCACTACGTGAACCATCACCCTAATCA
AGTTTTTTGGGGTCGAGGTGCCGTAAAGCACTAAATCGGAACCCTAAAGGGAGCCCCCGATTTAGAGCTTGACGG
GGAAAGCCGGCGAACGTGGCGAGAAAGGAAGGGAAGAAAGCGAAAGGAGCGGGCGCTAGGGCGCTGGCAAGTGTA
GCGGTCACGCTGCGCGTAACCACCACACCCGCCGCGCTTAATGCGCCGCTACAGGGCGCGTCCCATTCGCCATTC
AGGCTGCGCAACTGTTGGGAAGGGCGATCGGTGCGGGCCTCTTCGCTATTACGCCAGCTGGCGAAAGGGGGATGT
GCTGCAAGGCGATTAAGTTGGGTAACGCCAGGGTTTTCCCAGTCACGACGTTGTAAAACGACGGCCAGTGAGCGC
GCCTCGTTCATTCACGTTTTTGAACCCGTGGAGGACGGGCAGACTCGCGGTGCAAATGTGTTTTACAGCGTGATG
GAGCAGATGAAGATGCTCGACACGCTGCAGAACACGCAGCTAGATTAACCCTAGAAAGATAATCATATTGTGACG
TACGTTAAAGATAATCATGCGTAAAATTGACGCATGTGTTTTATCGGTCTGTATATCGAGGTTTATTTATTAATT
TGAATAGATATTAAGTTTTATTATATTTACACTTACATACTAATAATAAATTCAACAAACAATTTATTTATGTTT
ATTTATTTATTAAAAAAAAACAAAAACTCAAAATTTCTTCTATAAAGTAACAAAACTTTTATGAGGGACAGCCCC
CCCCCAAAGCCCCCAGGGATGTAATTACGTCCCTCCCCCGCTAGGGGGCAGCAGCGAGCCGCCCGGGGCTCCGCT
CCGGTCCGGCGCTCCCCCCGCATCCCCGAGCCGGCAGCGTGCGGGGACAGCCCGGGCACGGGGAAGGTGGCACGG
GATCGCTTTCCTCTGAACGCTTCTCGCTGCTCTTTGAGCCTGCAGACACCTGGGGGGATACGGGGAAAAGGCCTC
CAAGGCCACTAGGAAAAACCGCACTTGTCCGGAAACCCCGGGAATCTAACCCGGCTGAACGGATTTAGAGTCCGT
TCGATCTACATGATCAGGTTTCCGGTGTTTCGTCCTTTCCACAAGATATATAAAGCCAAGAAATCGAAATACTTT
CAAGTTACGGTAAGCATATGATAGTCCATTTTAAAACATAATTTTAAAACTGCAAACTACCCAAGAAATTATTAC
TTTCTACGTCACGTATTTTGTACTAATATCTTTGTGTTTACAGTCAAATTAATTCTAATTATCTCTCTAACAGCC
TTGTATCGTATATGCAAATATGAAGGAATCATGGGAAATAGGCCCTCTTCCTGCCCAACTAGGAAAAACCGCACT

TGTCCGGAAACCCCGGGAATCTAACCCGGCTGAACGGATTTAGAGTCCGTTCGATCTACATGATCAGGTTTCCGG
TGTTTCGTCCTTTCCACAAGATATATAAAGCCAAGAAATCGAAATACTTTCAAGTTACGGTAAGCATATGATAGT
CCATTTTAAAACATAATTTTAAAACTGCAAACTACCCAAGAAATTATTACTTTCTACGTCACGTATTTTGTACTA
ATATCTTTGTGTTTACAGTCAAATTAATTCTAATTATCTCTCTAACAGCCTTGTATCGTATATGCAAATATGAAG
GAATCATGGGAAATAGGCCCTCTTCCTGCCCAACTAGGAAAAACCGCACTTGTCCGGAAACCCCGGGAATCTAAC
CCGGCTGAACGGATTTAGAGTCCGTTCGATCTACATGATCAGGTTTCCGGTGTTTCGTCCTTTCCACAAGATATA
TAAAGCCAAGAAATCGAAATACTTTCAAGTTACGGTAAGCATATGATAGTCCATTTTAAAACATAATTTTAAAAC
TGCAAACTACCCAAGAAATTATTACTTTCTACGTCACGTATTTTGTACTAATATCTTTGTGTTTACAGTCAAATT
AATTCTAATTATCTCTCTAACAGCCTTGTATCGTATATGCAAATATGAAGGAATCATGGGAAATAGGCCCTCTTC
CTGCCCAACTAGGAAAAACCGCACTTGTCCGGAAACCCCGGGAATCTAACCCGGCTGAACGGATTTAGAGTCCGT
TCGATCTACATGATCAGGTTTCCGGTGTTTCGTCCTTTCCACAAGATATATAAAGCCAAGAAATCGAAATACTTT
CAAGTTACGGTAAGCATATGATAGTCCATTTTAAAACATAATTTTAAAACTGCAAACTACCCAAGAAATTATTAC
TTTCTACGTCACGTATTTTGTACTAATATCTTTGTGTTTACAGTCAAATTAATTCTAATTATCTCTCTAACAGCC
TTGTATCGTATATGCAAATATGAAGGAATCATGGGAAATAGGCCCTCTTCCTGCCCAActagtgaaaaaccgcac
ttgtccggaaaccccgggaatctaacccggctgaacggatttagagtccgttcgatctacatgatcaggtttccg
aggtacccaggcggcgcacaagctatataaacctgaaggaaatctcaactttacacttaggtcaagttacttatc
gtactagagcttcagcaggaaatttaactaaaatctaatttaaccagcatagcaaatatcatttattcccaaaat
gctaaagtttgagataaacggacttgatttccggctgttttgacactatccagaatgccttgcagatgggtgggg
catgctaaatactgcaggaaaaaccgcacttgtccggaaaccccgggaatctaacccggctgaacggatttagag
tccgttcgatctacatgatcaggtttccgaggtacccaggcggcgcacaagctatataaacctgaaggaaatctc
aactttacacttaggtcaagttacttatcgtactagagcttcagcaggaaatttaactaaaatctaatttaacca
gcatagcaaatatcatttattcccaaaatgctaaagtttgagataaacggacttgatttccggctgttttgacac
tatccagaatgccttgcagatgggtggggcatgctaaatactgcaggaaaaaccgcacttgtccggaaaccccgg
gaatctaacccggctgaacggatttagagtccgttcgatctacatgatcaggtttccgaggtacccaggcggcgc
acaagctatataaacctgaaggaaatctcaactttacacttaggtcaagttacttatcgtactagagcttcagca
ggaaatttaactaaaatctaatttaaccagcatagcaaatatcatttattcccaaaatgctaaagtttgagataa
acggacttgatttccggctgttttgacactatccagaatgccttgcagatgggtggggcatgctaaatactgcag
gaaaaaccgcacttgtccggaaaccccgggaatctaacccggctgaacggatttagagtccgttcgatctacatg
atcaggtttccgaggtacccaggcggcgcacaagctatataaacctgaaggaaatctcaactttacacttaggtc
aagttacttatcgtactagagcttcagcaggaaatttaactaaaatctaatttaaccagcatagcaaatatcatt
tattcccaaaatgctaaagtttgagataaacggacttgatttccggctgttttgacactatccagaatgccttgc
agatgggtggggcatgctaaatactgcagaaCTAGTGGATCTGCGATCGCTCCGGTGCCCGTCAGTGGGCAGAGC
GCACATCGCCCACAGTCCCCGAGAAGTTGGGGGGAGGGGTCGGCAATTGAACGGGTGCCTAGAGAAGGTGGCGCG
GGGTAAACTGGGAAAGTGATGTCGTGTACTGGCTCCGCCTTTTTCCCGAGGGTGGGGGAGAACCGTATATAAGTG
CAGTAGTCGCCGTGAACGTTCTTTTTCGCAACGGGTTTGCCGCCAGAACACAGCTGAAGCTTCGAGGGGCTCGCA
TCTCTCCTTCACGCGCCCGCCGCCCTACCTGAGGCCGCCATCCACGCCGGTTGAGTCGCGTTCTGCCGCCTCCCG
CCTGTGGTGCCTCCTGAACTGCGTCCGCCGTCTAGGTAAGTTTAAAGCTCAGGTCGAGACCGGGCCTTTGTCCGG
CGCTCCCTTGGAGCCTACCTAGACTCAGCCGGCTCTCCACGCTTTGCCTGACCCTGCTTGCTCAACTCTACGTCT
TTGTTTCGTTTTCTGTTCTGCGCCGTTACAGATCCAAGCTGTGACCGGCGCCTACTCTAGAGCTAGCGTTTAAAC
TTAAGAAGCTTGCCACCATGGACTACAAGGACGACGACGACAAGATGGACAAGAAGCCCCTGAACACCCTGATCA
GCGCCACAGGACTGTGGATGTCCAGAACCGGCACCATCCACAAGATCAAGCACCACGAGGTGTCCCGGTCCAAAA
TCTACATCGAGATGGCCTGCGGCGATCACCTGGTCGTCAACAACAGCAGAAGCAGCCGGACAGCCAGAGCCCTGC
GGCACCACAAGTACAGAAAGACCTGCAAGCGGTGCAGAGTGTCCGACGAGGACCTGAACAAGTTCCTGACCAAGG
CCAACGAGGACCAGACCAGCGTGAAAGTGAAGGTGGTGTCCGCCCCCACCCGGACCAAGAAAGCCATGCCCAAGA
GCGTGGCCAGAGCCCCCAAGCCCCTGGAAAACACCGAAGCCGCTCAGGCCCAGCCCAGCGGCAGCAAGTTCAGCC
CCGCCATCCCCGTGTCTACCCAGGAAAGCGTCAGCGTCCCCGCCAGCGTGTCCACCAGCATCTCTAGCATCTCAA
CCGGCGCCACAGCTTCTGCCCTGGTCAAGGGCAACACCAACCCCATCACCAGCATGTCTGCCCCTGTGCAGGCCT
CTGCCCCAGCCCTGACCAAGTCCCAGACCGACCGGCTGGAAGTGCTCCTGAACCCCAAGGACGAGATCAGCCTGA
ACAGCGGCAAGCCCTTCCGGGAGCTGGAAAGCGAGCTGCTGAGCCGGCGGAAGAAGGACCTCCAGCAAATCTACG
CCGAGGAACGGGAGAACTACCTGGGCAAGCTGGAAAGAGAGATCACCCGGTTCTTCGTGGACCGGGGCTTCCTGG
AAATCAAGAGCCCCATCCTGATCCCCCTGGAGTACATCGAGCGGATGGGCATCGACAACGACACCGAGCTGAGCA
AGCAGATTTTCCGGGTGGACAAGAACTTCTGCCTGCGGCCCATGCTGGCCCCCAACCTGTACAACTACCTGCGGA
AACTGGATCGCGCTCTGCCCGACCCCATCAAGATTTTCGAGATCGGCCCCTGCTACCGGAAAGAGAGCGACGGCA
AAGAGCACCTGGAAGAGTTTACAATGCTGAACTTTTGCCAGATGGGCAGCGGCTGCACCAGAGAGAACCTGGAAT
CCATCATCACCGACTTTCTGAACCACCTGGGGATCGACTTCAAGATCGTGGGCGACAGCTGCATGGTGTACGGCG
ACACCCTGGACGTGATGCACGGCGACCTGGAACTGTCTAGCGCCGTCGTGGGACCCATCCCTCTGGACCGGGAGT
GGGGCATCGATAAGCCCTGGATCGGAGCCGGCTTCGGCCTGGAACGGCTGCTGAAAGTCAAGCACGACTTTAAGA
ACATCAAGCGGGCTGCCAGAAGCGAGAGCTACTACAACGGCATCAGCACCAACCTGTGATGAGGATCCGCGGCCG
CGCCCCTCTCCCTCCCCCCCCCCTAACGTTACTGGCCGAAGCCGCTTGGAATAAGGCCGGTGTGCGTTTGTCTAT
ATGTTATTTTCCACCATATTGCCGTCTTTTGGCAATGTGAGGGCCCGGAAACCTGGCCCTGTCTTCTTGACGAGC
ATTCCTAGGGGTCTTTCCCCTCTCGCCAAAGGAATGCAAGGTCTGTTGAATGTCGTGAAGGAAGCAGTTCCTCTG

GAAGCTTCTTGAAGACAAACAACGTCTGTAGCGACCCTTTGCAGGCAGCGGAACCCCCCACCTGGCGACAGGTGC
CTCTGCGGCCAAAAGCCACGTGTATAAGATACACCTGCAAAGGCGGCACAACCCCAGTGCCACGTTGTGAGTTGG
ATAGTTGTGGAAAGAGTCAAATGGCTCTCCTCAAGCGTATTCAACAAGGGGCTGAAGGATGCCCAGAAGGTACCC
CATTGTATGGGATCTGATCTGGGGCCTCGGTGCACATGCTTTACATGTGTTTAGTCGAGGTTAAAAAAACGTCTA
GGCCCCCCGAACCACGGGGACGTGGTTTTCCTTTGAAAAACACGATGATAATATGGCCACAACCATGGCGTCCGG
AATGACCGAGTACAAGCCCACGGTGCGCCTCGCCACCCGCGACGACGTCCCCAGGGCCGTACGCACCCTCGCCGC
CGCGTTCGCCGACTACCCCGCCACGCGCCACACCGTCGATCCGGACCGCCACATCGAGCGGGTCACCGAGCTGCA
AGAACTCTTCCTCACGCGCGTCGGGCTCGACATCGGCAAGGTGTGGGTCGCGGACGACGGCGCCGCGGTGGCGGT
CTGGACCACGCCGGAGAGCGTCGAAGCGGGGGCGGTGTTCGCCGAGATCGGCCCGCGCATGGCCGAGTTGAGCGG
TTCCCGGCTGGCCGCGCAGCAACAGATGGAAGGCCTCCTGGCGCCGCACCGGCCCAAGGAGCCCGCGTGGTTCCT
GGCCACCGTCGGCGTCTCGCCCGACCACCAGGGCAAGGGTCTGGGCAGCGCCGTCGTGCTCCCCGGAGTGGAGGC
GGCCGAGCGCGCCGGGGTGCCCGCCTTCCTGGAGACCTCCGCGCCCCGCAACCTCCCCTTCTACGAGCGGCTCGG
CTTCACCGTCACCGCCGACGTCGAGGTGCCCGAAGGACCGCGCACCTGGTGCATGACCCGCAAGCCCGGTGCCTA
GGTCGACAATCAACCTCTGGATTACAAAATTTGTGAAAGATTGACTGGTATTCTTAACTATGTTGCTCCTTTTAC
GCTATGTGGATACGCTGCTTTAATGCCTTTGTATCATGTTAACTTGTTTATTGCAGCTTATAATGGTTACAAATA
AAGCAATAGCATCACAAATTTCACAAATAAAGCATTTTTTTCACTGCATTCTAGTTGTGGTTTGTCCAAACTCAT
CAATGTATCTTATCATGTCTGGAATTGACTCAAATGATGTCAATTAGTCTATCAGAAGCTATCTGGTCTCCCTTC
CGGGGGACAAGACATCCCTGTTTAATATTTAAACAGCAGTGTTCCCAAACTGGGTTCTTATATCCCTTGCTCTGG
TCAACCAGGTTGCAGGGTTTCCTGTCCTCACAGGAACGAAGTCCCTAAAGAAACAGTGGCAGCCAGGTTTAGCCC
CGGAATTGACTGGATTCCTTTTTTAGGGCCCATTGGTATGGCTTTTTCCCCGTATCCCCCCAGGTGTCTGCAGGC
TCAAAGAGCAGCGAGAAGCGTTCAGAGGAAAGCGATCCCGTGCCACCTTCCCCGTGCCCGGGCTGTCCCCGCACG
CTGCCGGCTCGGGGATGCGGGGGGAGCGCCGGACCGGAGCGGAGCCCCGGGCGGCTCGCTGCTGCCCCCTAGCGG
GGGAGGGACGTAATTACATCCCTGGGGGCTTTGGGGGGGGGCTGTCCCTGATATCTATAACAAGAAAATATATAT
ATAATAAGTTATCACGTAAGTAGAACATGAAATAACAATATAATTATCGTATGAGTTAAATCTTAAAAGTCACGT
AAAAGATAATCATGCGTCATTTTGACTCACGCGGTCGTTATAGTTCAAAATCAGTGACACTTACCGCATTGACAA
GCACGCCTCACGGGAGCTCCAAGCGGCGACTGAGATGTCCTAAATGCACAGCGACGGATTCGCGCTATTTAGAAA
GAGAGAGCAATATTTCAAGAATGCATGCGTCAATTTTACGCAGACTATCTTTCTAGGGTTAATCTAGCTGCATCA
GGATCATATCGTCGGGTCTTTTTTCCGGCTCAGTCATCGCCCAAGCTGGCGCTATCTGGGCATCGGGGAGGAAGA
AGCCCGTGCCTTTTCCCGCGAGGTTGAAGCGGCATGGAAAGAGTTTGCCGAGGATGACTGCTGCTGCATTGACGT
TGAGCGAAAACGCACGTTTACCATGATGATTCGGGAAGGTGTGGCCATGCACGCCTTTAACGGTGAACTGTTCGT
TCAGGCCACCTGGGATACCAGTTCGTCGCGGCTTTTCCGGACACAGTTCCGGATGGTCAGCCCGAAGCGCATCAG
CAACCCGAACAATACCGGCGACAGCCGGAACTGCCGTGCCGGTGTGCAGATTAATGACAGCGGTGCGGCGCTGGG
ATATTACGTCAGCGAGGACGGGTATCCTGGCTGGATGCCGCAGAAATGGACATGGATACCCCGTGAGTTACCCGG
CGGGCGCGCTTGGCGTAATCATGGTCATAGCTGTTTCCTGTGTGAAATTGTTATCCGCTCACAATTCCACACAAC
ATACGAGCCGGAAGCATAAAGTGTAAAGCCTGGGGTGCCTAATGAGTGAGCTAACTCACATTAATTGCGTTGCGC
TCACTGCCCGCTTTCCAGTCGGGAAACCTGTCGTGCCAGCTGCATTAATGAATCGGCCAACGCGCGGGGAGAGGC
GGTTTGCGTATTGGGCGCTCTTCCGCTTCCTCGCTCACTGACTCGCTGCGCTCGGTCGTTCGGCTGCGGCGAGCG
GTATCAGCTCACTCAAAGGCGGTAATACGGTTATCCACAGAATCAGGGGATAACGCAGGAAAGAACATGTGAGCA
AAAGGCCAGCAAAAGGCCAGGAACCGTAAAAAGGCCGCGTTGCTGGCGTTTTTCCATAGGCTCCGCCCCCCTGAC
GAGCATCACAAAAATCGACGCTCAAGTCAGAGGTGGCGAAACCCGACAGGACTATAAAGATACCAGGCGTTTCCC
CCTGGAAGCTCCCTCGTGCGCTCTCCTGTTCCGACCCTGCCGCTTACCGGATACCTGTCCGCCTTTCTCCCTTCG
GGAAGCGTGGCGCTTTCTCATAGCTCACGCTGTAGGTATCTCAGTTCGGTGTAGGTCGTTCGCTCCAAGCTGGGC
TGTGTGCACGAACCCCCCGTTCAGCCCGACCGCTGCGCCTTATCCGGTAACTATCGTCTTGAGTCCAACCCGGTA
AGACACGACTTATCGCCACTGGCAGCAGCCACTGGTAACAGGATTAGCAGAGCGAGGTATGTAGGCGGTGCTACA
GAGTTCTTGAAGTGGTGGCCTAACTACGGCTACACTAGAAGGACAGTATTTGGTATCTGCGCTCTGCTGAAGCCA
GTTACCTTCGGAAAAAGAGTTGGTAGCTCTTGATCCGGCAAACAAACCACCGCTGGTAGCGGTGGTTTTTTTGTT
TGCAAGCAGCAGATTACGCGCAGAAAAAAAGGATCTCAAGAAGATCCTTTGATCTTTTCTACGGGGTCTGACGCT
CAGTGGAACGAAAACTCACGTTAAGGGATTTTGGTCATGAGATTATCAAAAAGGATCTTCACCTAGATCCTTTTA
AATTAAAAATGAAGTTTTAAATCAATCTAAAGTATATATGAGTAAACTTGGTCTGACAGTTACCAATGCTTAATC
AGTGAGGCACCTATCTCAGCGATCTGTCTATTTCGTTCATCCATAGTTGCCTGACTCCCCGTCGTGTAGATAACT
ACGATACGGGAGGGCTTACCATCTGGCCCCAGTGCTGCAATGATACCGCGAGACCCACGCTCACCGGCTCCAGAT
TTATCAGCAATAAACCAGCCAGCCGGAAGGGCCGAGCGCAGAAGTGGTCCTGCAACTTTATCCGCCTCCATCCAG
TCTATTAATTGTTGCCGGGAAGCTAGAGTAAGTAGTTCGCCAGTTAATAGTTTGCGCAACGTTGTTGCCATTGCT
ACAGGCATCGTGGTGTCACGCTCGTCGTTTGGTATGGCTTCATTCAGCTCCGGTTCCCAACGATCAAGGCGAGTT
ACATGATCCCCCATGTTGTGCAAAAAAGCGGTTAGCTCCTTCGGTCCTCCGATCGTTGTCAGAAGTAAGTTGGCC
GCAGTGTTATCACTCATGGTTATGGCAGCACTGCATAATTCTCTTACTGTCATGCCATCCGTAAGATGCTTTTCT
GTGACTGGTGAGTACTCAACCAAGTCATTCTGAGAATAGTGTATGCGGCGACCGAGTTGCTCTTGCCCGGCGTCA
ATACGGGATAATACCGCGCCACATAGCAGAACTTTAAAAGTGCTCATCATTGGAAAACGTTCTTCGGGGCGAAAA
CTCTCAAGGATCTTACCGCTGTTGAGATCCAGTTCGATGTAACCCACTCGTGCACCCAACTGATCTTCAGCATCT
TTTACTTTCACCAGCGTTTCTGGGTGAGCAAAAACAGGAAGGCAAAATGCCGCAAAAAAGGGAATAAGGGCGACA
CGGAAATGTTGAATACTCAT
